# Supplementary figures and images for: Effectiveness of FIFA injury prevention programs in reducing ankle injuries among football players: a systematic review
Source: PeerJ. 2025 Mar 10;13:e18910. doi: 10.7717/peerj.18910 (PMC11905912; doi:10.7717/peerj.18910)

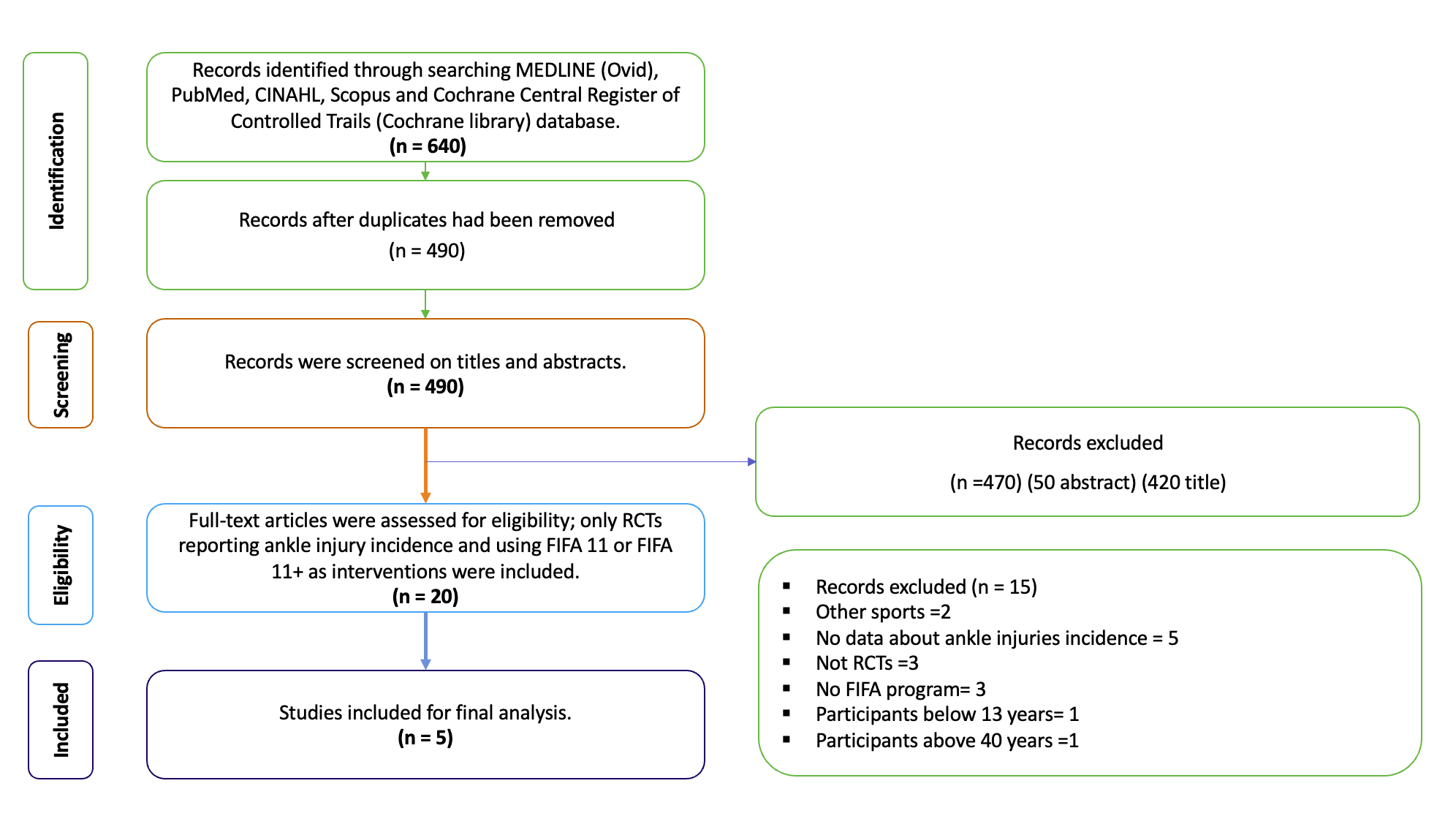

Supplement: Figure S1 [file peerj-13-18910-s001.png]
