# Supplementary material for: Effectiveness of FIFA injury prevention programs in reducing ankle injuries among football players: a systematic review
Source: PeerJ. 2025 Mar 10;13:e18910. doi: 10.7717/peerj.18910 (PMC11905912; doi:10.7717/peerj.18910)
Supplement: Supplemental Information 2 [file peerj-13-18910-s002.pdf]

The Rationale for Conducting the Systematic Review/Meta-Analysis:

It is widely recognized that the FIFA injury prevention programme are comprehensive programmes that help to avoid overall football injuries among footballers (Al Attar *et al.*, 2016). The majority of previous studies have highlighted how the FIFA 11+ programme can reduce football injuries to the lower and upper limbs as well as overall injury incidence rates (Mayo, Seijas & Álvarez, 2014). While research has shown the programme to be effective in reducing general lower limb injuries, researchers have not provided enough evidence to help coaches decide which specific injuries (e.g. ankle or knee injuries) are best targeted by the FIFA 11+ programme (Mayo, Seijas & Álvarez, 2014).

There are several reasons why the author of this study chose to focus on ankle injuries in particular, instead of investigating how to reduce the overall number of football injuries. Firstly, a search of the previous literature revealed a significant gap in the research as no previous systematic reviews have focused on how the FIFA injury prevention programmes can help to prevent ankle injuries. Furthermore, in terms of injuries to the ankle, ankle sprains are the most common type (Fong *et al.*, 2007), therefore investigating a programme that may help to prevent them has great value in this field. It is clear that focusing on this type of injury will help to reduce the incidence of ankle injuries among football players, particularly those who have experienced these injuries in the past. Highlighting the effectiveness of the FIFA programmes will also hopefully motivate coaches and teams to incorporate it into their training regimes. A further reason for the author wishing to examine the effectiveness of the FIFA injury prevention programmes with regard to ankle injuries was the severity of these injuries for football players. It can take a long time for them to recover and be able to rejoin the team (Walls *et al.*, 2016). Lastly, included in the FIFA prevention programmes are exercises known as proprioception (balance) exercises (Al Attar *et al.*, 2016). These can help to prevent ankle injuries, and they therefore make the FIFA programmes interesting to study in terms of reducing the incidence of ankle injuries among sportsmen.

Furthermore, from the comprehensive search (literature review search) there is scant literature focusing on the effect of the FIFA prevention programmes on a specific part of

the body, such as the ankle joint or knee joint. Most of the literature seemed to be generally concerned with overall body injuries. The search revealed only one big study, conducted by Silvers-Granelli *et al.*, (2017) that assessed the effect of the FIFA 11+ prevention programme on the ACL (Anterior Cruciate Ligament) incidence injuries in male footballers. The study included 65 teams which were randomised into two groups. The intervention group involved 31 teams (675 players), while the control groups involved 34 teams (850 players). Footballers in the intervention group performed the FIFA 11+ programme, while those in the control group performed the usual of the teams (their normal training). Interestingly the results of this research indicated that the FIFA 11+ program diminished the overall occurrence percentage of ACL injury by 77%. Although, the study by Silvers-Granelli *et al.*, (2017) was conducted on only male football players. While the females are more vulnerable to an ACL injury (Montalvo *et al.*, 2019). This makes it hard to generalize the study on both genders.

Contribution to Knowledge:

This systematic review contributes to the existing body of knowledge by providing a focused evaluation of the FIFA injury prevention programs on ankle injuries, an area that has been underexplored in previous research. While other studies and meta-analyses have assessed the overall effectiveness of these programs in reducing injuries in football, none have specifically addressed their impact on ankle injuries. The findings of this review highlight the differential effectiveness of the FIFA 11 and FIFA 11+ programs, with the latter demonstrating a significant reduction in ankle injury rates. By distinguishing between the two programs and emphasizing the superior efficacy of FIFA 11+ in ankle injury prevention, this review adds valuable insights to the literature, offering evidence-based guidance for football teams and healthcare professionals aiming to mitigate ankle injuries. Additionally, this review underscores the importance of joint-specific injury prevention strategies within broader injury prevention programs, thereby informing future research and the development of more targeted interventions in sports injury prevention.

References

Al Attar WS, Soomro N, Pappas E, Sinclair PJ, Sanders RH. How Effective are F-MARC Injury Prevention Programs for Soccer Players? A Systematic Review and Meta-Analysis. Sports Med. 2016;46(2):205-217. doi:10.1007/s40279-015-0404-x

Mayo M, Seijas R, Alvarez P. Calentamiento neuromuscular estructurado como prevenci√≥n de lesiones en futbolistas profesionales j√≥venes [Structured neuromuscular warm-up for injury prevention in young elite football players]. Rev Esp Cir Ortop Traumatol. 2014;58(6):336-342. doi:10.1016/j.recot.2014.05.008

Fong DT, Hong Y, Chan LK, Yung PS, Chan KM. A systematic review on ankle injury and ankle sprain in sports. Sports Med. 2007;37(1):73-94. doi:10.2165/00007256-200737010-00006

Walls, R. J., Ross, K. A., Fraser, E. J., Hodgkins, C. W., Smyth, N. A., Egan, C. J., Calder, J. & Kennedy, J. G. (2016) 'Football injuries of the ankle: A review of injury mechanisms, diagnosis and management'. World journal of orthopedics, 7 (1), pp. 8.

Silvers-Granelli H, Mandelbaum B, Adeniji O, et al. Efficacy of the FIFA 11+ Injury Prevention Program in the Collegiate Male Soccer Player. Am J Sports Med. 2015;43(11):2628-2637. doi:10.1177/0363546515602009

Montalvo, A. M., Schneider, D. K., Webster, K. E., Yut, L., Galloway, M. T., Heidt, R. S., Jr., Kaeding, C. C., Kremcheck, T. E., Magnussen, R. A., Parikh, S. N., Stanfield, D. T., Wall, E. J. & Myer, G. D. (2019) 'Anterior Cruciate Ligament Injury Risk in Sport: A Systematic Review and Meta-Analysis of Injury Incidence by Sex and Sport Classification'. J Athl Train, 54 (5), pp. 472-482.
